# Correction: Ventilation Defect Formation in Healthy and Asthma Subjects Is Determined by Lung Inflation

**DOI:** 10.1371/annotation/0dafbfa4-2289-40ec-9f21-35b24a9302c3

**Published:** 2014-01-23

**Authors:** R. Scott Harris, Hanae Fujii-Rios, Tilo Winkler, Guido Musch, Marcos F. Vidal Melo, José G. Venegas

There is an error in Table 2. The entry cov squared sVdotA in the left-hand column should not be squared. 

The correct version of Table 2 can be viewed here: 

**Figure:**
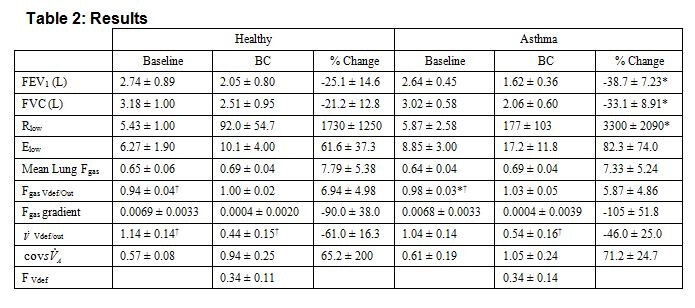


The correct version of the legend of Table 2 can be viewed here: http://www.plosone.org/corrections/pone.0053216.001.cn.tif

